# Notes on Preparations for the Sick

**Published:** 1894-12

**Authors:** 


### Notes on Preparations for the Sick.

**Fluid Cascara Aromatic. Hypodermic Tablets. Pepsin Aseptic, scale and powder.**—Parke, Davis & Co., London.—The **Fluid Cascara Aromatic** is more concentrated than the Cascara Cordial, and is more palatable than either that or the ordinary fluid extract or its tasteless form. This drug, originally introduced by Messrs. Parke, Davis & Co., still maintains its supremacy, and no preparations of it are better known or more trustworthy than are those of this firm. The aromatic fluid cascara is likely to be the favourite, and to be much in demand.

**Hypodermic Tablets** have established themselves as the most convenient agents for hypodermic use. The difficulty has been that many tablets are difficult to dissolve without hot water and waste of time; but these tablets are very soluble, and may be relied upon for accuracy of dose as well as therapeutic efficiency. They will keep in any climate, and are always ready for use. We have long given up the use of fluids for hypodermic use in favour of some kind of soluble tablet.

The **Aseptic Pepsins,** in scale or powder, are completely soluble, and will digest four thousand times their weight of albumen. They are almost free from odour and taste, and are manifestly superior to the pepsines which have previously been in use. So many kinds of pepsines are now in the market that it becomes necessary to specify which variety is needed. None are better than those of a firm which has an established reputation to maintain.

---

**Cerebos Salt.**—The Cerebos Salt Co. Ltd., Newcastle-on-Tyne.—This is a nutritive table salt, being composed of a mixture of common salt with phosphates. If mankind lived on bread only, and that bread were white, then some such salt as this would be needful to supply the deficient phosphates, but with a mixed diet we cannot assert that this salt is a necessity for perfect nutrition: but it is a fine, dry, excellent salt for table purposes; it does not cake even in the dampest weather, it answers every purpose for which table salt is used, and the phosphates present are likely to be of some little utility. We have used it for several weeks, and have every reason to look upon it as an improvement.

---

**Californian Wines: No. 1, Claret Type; No. 3, Burgundy; No. 12, Burgundy; No. 5, Chablis; No. 6, Sauterne; No. 7, Hock.**—Grierson, Oldham & Co., London.—We have given these wines a trial, and we like them exceedingly. They are made from the same variety of grapes as in Europe; but in California they develop somewhat different characteristics. For instance, the white wines made from the Riesling, or Hock grapes, although resembling in many respects the German Hocks, have very much less acidity. and, as a rule, more body; this is owing, no doubt, to the fact that these grapes thoroughly ripen in California, whereas in Germany they scarcely ever do so. The wines of Burgundy type are excellent and of moderate price. No. 5 is a clean dry wine, of Chablis type, but with much less acidity than is usual. The white wine, No. 7, made from the Johannisberg Riesling grape, is a high-class hock, of good alcoholic strength, free from acidity, and having considerable flavour and bouquet. Messrs. R. W. Miller & Co., Stokes Croft, are the local agents.

---

**"Johannis."**—THE JOHANNIS COMPANY LIMITED, London.—We can speak in the highest terms of this exceedingly pleasant table water, samples of which have been sent to us. It is now so well known and its virtues are so widely recognised that it is not necessary to refer to them in detail.

---

**Diaphtherin.** — FARBWERKE VORM. MEISTER LUCIUS & BRUENING, Hoechst o-Main.—We have received from Messrs. Burroughs, Wellcome & Co., who are the sole agents for Great Britain and Ireland, a sample of this preparation, which is a sulphur-yellow powder of slight odour possessed of powerful antiseptic properties rendering it an excellent substitute for iodoform. It is not poisonous and has no caustic action, but is very soluble in water. We have been very pleased with its action in the two or three cases in which we have tried it. It may be freely used in the mouth or nasal cavities.

---

**Europhen. Stoddart's Soluble Sea Salts.**—A. & J. WARREN, Bristol.—**Europhen** is an iodised substitution product of iso-butyl-cresol, and may be employed in place of iodoform. Used as a dusting powder as a dry antiseptic, or in solution in olive oil, it has given good results, and being of light weight its drying power is great, whilst it is comparatively odourless and not poisonous.

**Stoddart's Sea Salts** furnish a ready method for the instantaneous preparation of sea water for bathing purposes: a large teacupful readily dissolves in each gallon of hot or cold water. These salts have been in use for many years, and have a well-established reputation.

---

**Hazeline. Pinol. Compound Menthol Snuff. Ichthyol Ointment.**—BURROUGHS, WELLCOME & Co., London.—We have received a specimen of **Hazeline,** the valuable properties of which are now so well known that it needs no praise at our hands; and also a specimen of **Pinol,** which is a fragrant volatile oil of exquisite fragrance, useful for internal administration in doses of two to five minims, as well as for inhalation. **Compound Menthol Snuff** contains a small quantity of cocaine, and is useful in hay asthma, as well as in various forms of rhinitis. **Ichthyol Ointment** is a somewhat potent remedy, intended for application to *unbroken* inflamed surfaces; it is conveniently supplied in metallic tubes for easy use.

---

**Olive Oil Soap. Pure Carbolic Soap.**—CHRISTOPHER THOMAS & BROS., Limited, Bristol.—These two specimens of soap have been forwarded as samples of the products of an old Bristol firm, which has maintained its position notwithstanding the energy and activity of its young and enterprising advertising competitors.

The **Olive Oil Soap** is made from a recipe which was first used by the firm's predecessors, Messrs. Saml. Fripp & Co., some century and a half ago. Fripp's Olive Oil Soap has had a long-lived reputation, and is even now by no means out of date. It is a good nursery and toilet soap, and the firm has frequently received testimonials as to its efficacy in relieving the irritation caused by eczema and similar skin diseases. This soap must still be classed in the first rank, as it is pleasant to use, and has nothing irritating or objectionable in its composition. The **Carbolic Soap** contains five per cent. Calvert's carbolic white crystals.

---

**Cellular Cloth.**—THE CELLULAR CLOTHING COMPANY LTD., London.—Mr. G. Standerwick, of Stokes Croft, who is the local agent, has sent us some samples of this material, the special qualities of which are:—1. It retains warmth, but yet permits free evaporation. 2. Owing to the retention of air (the best non-conductor of heat) in the cells of the cloth, it is warm in winter and cool in summer. 3. Being exceptionally porous, it is the cleanliest and most easily washed of all forms of clothing. 4. It is extremely durable. 5. The question of material (cotton, wool, or silk) becomes of secondary importance; the retention of air in the cells determines the warmth. Patterns in wool, cotton, and silk, with various mixtures of material, have been forwarded to us: we can only say that they appear to be of excellent make, and give a very large variety of pattern. The various garments devised by the Cellular Clothing Company are arranged to suit all sorts and conditions of men, women, and children: the only remark we need make is that in recommending our patients to wear them we are adopting the usual method of medical self-abnegation, inasmuch as we are told that "the wearers of cellular clothing are remarkably free from colds and chills." This being the case, we need such protection even more than many of our patients, and we may at least set them an excellent example. From personal experience we are able to say that the pyjama suits are exceedingly comfortable.

